# Long-term treatment with budesonide/formoterol attenuates circulating CRP levels in chronic obstructive pulmonary disease patients of group D

**DOI:** 10.1371/journal.pone.0183300

**Published:** 2017-08-23

**Authors:** Yi-Hua Lin, Xi-Ning Liao, Li-Li Fan, Yue-Jin Qu, De-Yun Cheng, Yong-Hong Shi

**Affiliations:** 1 Department of Respiratory Medicine, the First Affiliated Hospital of Xiamen University, Xiamen, Fujian, China; 2 Department of Respiratory Medicine, West China Hospital of Sichuan University, Chengdu, Sichuan, China; UCLA, UNITED STATES

## Abstract

**Background:**

The systemic inflammation is associated with clinical outcome and mortality in chronic obstructive pulmonary disease (COPD) patients. To investigate the effects of tiotropium (Tio) and/or budesonide/formoterol (Bud/Form) on systemic inflammation biomarkers in stable COPD patients of group D, a randomized, open-label clinical trial was conducted.

**Methods:**

Eligible participants (n = 324) were randomized and received either Tio 18ug once daily (group I), Bud/Form 160/4.5ug twice daily (group II), Bud/Form 320/9ug twice daily (group III), or Tio 18ug once daily with Bud/Form 160/4.5ug twice daily (group IV) for 6 months. Systemic inflammation biomarkers were measured before randomization and during the treatment, including C-reactive protein (CRP), interleukin-6 (IL-6), interleukin-8 (IL-8), serum amyloid A (SAA), tumor necrosis factor-α (TNF-α), fibrinogen (Fib), and white blood cell (WBC).

**Results:**

After 6-month treatment, CRP levels in group II, group III and group IV changed by a median (interquartile range) of -1.25 (-3.29, 1.18) mg/L, -1.13 (-2.55, 0.77) mg/L, and -1.56 (-4.64, 0.22) mg/L respectively, all of which with statistical differences compared with group I. In addition, there were no treatment differences in terms of IL-8, SAA, TNF-α, Fib and WBC levels.

**Conclusions:**

A long-term treatment with Bud/Form alone or together with Tio can attenuate circulating CRP levels in COPD patients of group D, compared with Tio alone.

## Introduction

Chronic obstructive pulmonary disease (COPD) is one of the leading causes of disability and death worldwide. It is the fourth most common cause of death in the world[1], and it is predicted to rise to the third place by the year of 2020[2].Increasing studies have shown that COPD is not only a respiratory inflammatory condition[3], but also a mild chronic systemic inflammation accompanied by many extrapulmonary manifestations[4]. Several biomarkers of systemic inflammation have been proved to be directly associated with COPD, including C-reactive protein (CRP), interleukin-6 (IL-6), interleukin-8 (IL-8), serum amyloid A (SAA), tumor necrosis factor-α (TNF-α), fibrinogen (Fib), and white blood cell (WBC). The systemic inflammation is also related with lung function, arterial oxygen tension, exercise capacity, degree of dyspnea, clinical outcome, risk of exacerbation, all-cause and COPD-related mortality[5–7]. Moreover, the systemic inflammation could increase the risk of major comorbidities in COPD, including cardiovascular disease, lung cancer, pneumonia, diabetes mellitus, depression, cachexia, skeletal muscle dysfunction and osteoporosis[8–10]. Take all the factors above into consideration, attenuating systemic inflammation might provide a potential method to improve health conditions and prognosis in COPD patients. Inhaled corticosteroid (ICS) +long-acting β2-agonist (LABA) and/or long-acting anticholinergic agents (LAMA) reduce exacerbation risks and symptoms in COPD patients. Nevertheless, the effects of inhaled therapy on systemic inflammation in COPD are still controversial[11–14].

Many clinical symptoms and a high risk of exacerbations were observed in COPD patients of group D[15], whose CRP values were significantly higher than those in the other three groups[16]. Thus, a clinical trial was conducted to investigate the effects of tiotropium (Tio) and/or budesonide/formoterol (Bud/Form) on systemic inflammation biomarkers in stable COPD patients of group D and improvements of symptoms and pulmonary function.

## Methods

This randomized, open-label study aimed to evaluate the effect of inhaled therapy on systemic inflammation biomarkers in stable COPD patients of group D over 6 months. The study protocol was in compliance with the Declaration of Helsinki and was approved by the ethical committee of GCP and biomedicine of West China Hospital of Sichuan University. Written informed consents were obtained from all subjects. The study was registered at http://www.chictr.org.cn/ (ChiCTR-IPR-14005619).

### Study subjects

Outpatients with a clinical diagnosis of stable COPD were recruited from Jan 2015 to Nov 2015 in the West China Hospital of Sichuan University, China. The inclusion criteria were as follows: confirmed COPD of group D by pulmonary physicians based on the 2011 GOLD guidelines[15], age ≥40 years, absence of exacerbations[15] for at least one month. Exclusion criteria: patients used LAMA, LABA, oral corticosteroid or ICS in the previous one month, with any infection in the previous one month before study entry, with proved prostatic hyperplasia, bladder neck stenosis or narrow angle glaucoma, with other clinically significant lung diseases or complications which were associated with increasing systemic inflammation (e.g. rheumatoid arthritis, hepatic diseases, renal diseases, cancer or tuberculosis).

### Study design

Eligible participants were then randomly assigned (1:1:1:1) using a computer-generated randomization list to one of four arms: group I, Tio 18ug once daily by Handihaler (Boehringer Ingelheim Pharma, Ingelheim, Germany); group II, Bud/Form 160/4.5ug/dose (Symbicort Turbuhaler; AstraZeneca, Sweden) one inhalation twice daily; group III, Bud/Form 160/4.5ug/dose two inhalations twice daily; group IV, Tio 18ug once daily plus Bud/Form 160/4.5ug/dose one inhalation twice daily. All subjects were allowed to take Terbutaline (Bricasol pressurized metered-dose inhale; AstraZeneca, Wuxi, China) as reliever during the study period. No other bronchodilator was permitted to be used throughout the study. Considering life style modifications may have impacts on the level of systemic inflammation, specific instructions on risk-factor modifications were not given during the study, such as exercise training and smoking cessation.

The treatment duration was 6 months, with clinical visits at the end of the first month (visit 2), the third month (visit 3) and the sixth month (visit 4). During the treatment, subjects with any of the following conditions would be removed from the study: poor compliance (taking <80% or >120% of the dosage); combined with acute exacerbations; occurring serious adverse events; combined with any one of the above exclusion criteria.

### Outcome measurements

During each visit, fasting blood samples were collected in the morning. The samples were stored at -80°C until analyzed with standard hospital assays in the central laboratory. Whole blood leukocyte counts were measured using Sysmex XE-2100 automated hematology analyzer (Sysmex Medical Electronics Co., Ltd, Japan). CRP levels were determined using high-sensitivity immunoturbidimetric assay (Beckman Coulter, Inc, USA). IL-6 levels were measured by electrochemiluminescence assay (Roche Diagnostics Co., Ltd, Switzerland). IL-8 and TNF-α levels were measured by chemiluminescence immunoassay, whereas SAA serum concentrations were determined by nephelometry assay (Siemens Medical Diagnostic Products Co., Ltd, Germany). Fibrinogen levels were measured using coagulation method (Sysmex Medical Electronics Co., Ltd, Japan). All samples were analyzed in duplicate.

In addition, at each visit, participants’ symptoms were evaluated according to the modified British medical Research Council (mMRC) dyspnea scale[17] and COPD assessment test (CAT)[18]. Besides, at visit 1, 3 and 4, spirometry was performed [19].

### Statistical analysis

CRP was selected as the reference for calculation of the estimated sample size. It was assumed that the mean difference in the change of CRP level was 1.5 mg/L between groups with an SD of 3 mg/L[14], the level of significance was 0.05, the power of the test was 0.8, the lost follow-up rate was no more than 20%, and the calculated sample size was 80 per treatment group.

All statistical analysis was performed using IBM SPSS Statistics for Windows, version 19.0, Armonk, NY: IBM Corp. Analyses were conducted based on an intention-to-treat principle. Normally distributed data were described as mean ± standard deviations (SD), whereas non-normally distributed data were reported as medians (interquartile range, IQR) unless otherwise indicated.

For each visit of every group, changes from baseline in systemic inflammation biomarkers, lung function, and symptom scores were analyzed using paired *t* tests or Wilcoxon signed rank test, according to whether they meet the normal distribution.

For normally distributed variables, the differences of changes (post-treatment minus pre-treatment) of IL-8 levels, TNF-α levels, Fib levels, WBC levels, forced expiratory volume in one second (FEV_1_), forced vital capacity (FVC), and CAT scores between each groups were analyzed using an analysis of variance (ANOVA) model. And the least significance difference test was applied for pairwise comparison following ANOVA. For non-normally distributed variables, the differences of changes (post-treatment minus pre-treatment) of CRP levels, IL-6 levels, and SAA levels between each group were analyzed using Kruskal-Wallis test. And the Mann-Whitney U test applying for pairwise comparison was followed.

## Results

**Fig 1:**
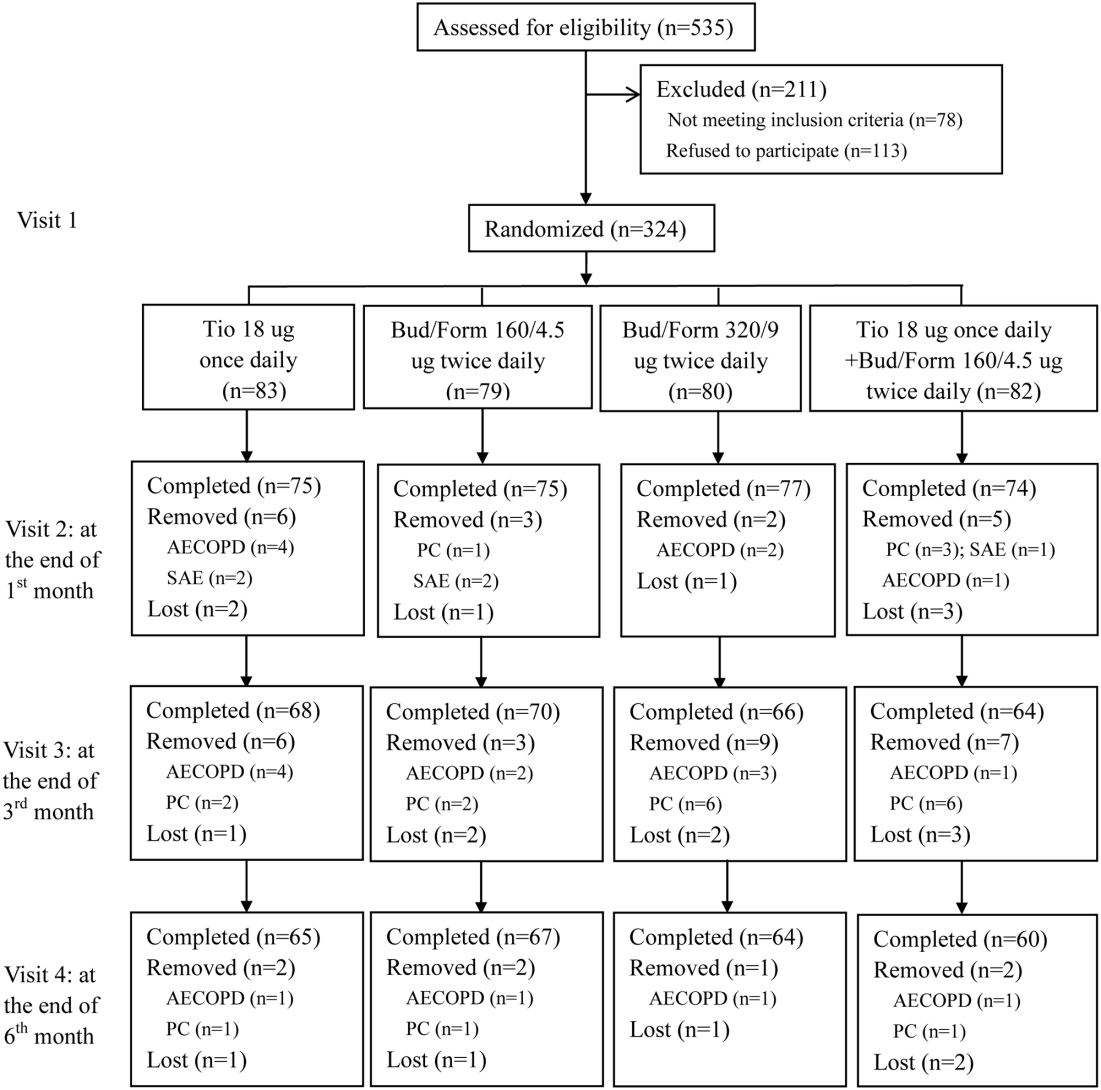
Flow diagram of patients in the study. Tio, tiotropium; Bud, budesonide; Form, formoterol; AECOPD, acute exacerbation of chronic obstructive pulmonary disease; SAE, serious adverse events; PC, poor compliance.

A total of 324 subjects were eligible for randomization. Among them, 301 subjects (92.9%) completed the first visit after 1 month of treatment, 268 subjects (82.7%) completed the second visit after 3 months of treatment, and 256 subjects (79%) completed the third visit after 6 months of treatment (Fig 1). Baseline characteristics of the four groups were similar (Table 1).

**Table 1:** Baseline characteristics of the study subjects.

	Group I(n = 83)	Group II(n = 79)	Group III(n = 80)	Group IV(n = 82)	*P*
Male (%)	79.5	69.6	73.8	74.4	0.958
Age (years)	65.31±9.75	65.27±9.57	66.44±9.82	63.45±9.90	0.274
Non smokers	15	16	21	19	0.974
Pack-years^δ^	30(10,40)	30(10,42)	25(5,40)	30(3,40)	0.668
BMI(kg/m^2^)	20.89±2.42	21.44±2.16	21.53±2.52	21.92±2.75	0.066
Duration COPD years^δ^	7(2, 15)	7(1, 10)	5(1.25, 10)	5.5(1, 10)	0.778
Exacerbations*^δ^	2(1, 2)	2(1,2)	2(1, 2)	1(1, 2)	0.340
Hospitalizations*	0.16±0.43	0.22±0.47	0.20±0.46	0.15±0.39	0.676
FEV_1_ (L)	0.88±0.34	0.82±0.27	0.93±0.27	0.81±0.30	0.709
FEV_1_% predicted	37.73±9.97	39.08±10.78	42.41±12.43	34.12±12.85	0.295
FVC (L)	2.21±0.72	1.89±0.65	2.21±0.65	1.95±0.50	0.369
FEV_1_/FVC (%)	40.11±7.29	45.63±8.63	43.59±8.32	41.51±11.15	0.325
Cor Pulmonale	5	8	5	3	0.480
Comorbidities					
CVD	6	6	6	8	0.944
Hypertension	5	6	7	5	0.909
DM	9	5	4	5	0.547
mMRC					0.574
1	1	0	0	1	
2	20	26	28	20	
3	60	49	50	56	
4	2	4	2	5	
CAT score	16.29±5.14	16.15±4.74	15.40±4.19	15.77±4.91	0.633
CRP(mg/L)^δ^	3.54(1.60, 5.32)	3.90(2.97, 6.56)	3.69(1.98, 5.21)	4.20(2.36, 6.40)	0.197
IL-6(pg/mL)^δ^	4.40(3.13, 7.29)	4.50(3.09, 9.39)	4.55(3.11, 8.61)	5.34(3.06, 10.11)	0.527
IL-8(pg/mL)	11.54±6.82	10.99±5.61	10.44±4.61	11.41±5.84	0.616
SAA(mg/L)^δ^	3.52(2.32, 10.00)	4.06(2.32, 11.98)	4.12(2.66, 7.37)	4.46(2.42, 12.22)	0.728
TNF-α(pg/mL)	10.39±3.19	10.90±3.66	10.73±3.60	11.37±4.19	0.391
Fib(g/L)	3.58±0.90	3.49±0.76	3.50±0.84	3.49±0.85	0.898
WBC(×10^9^/L)	6.67±1.66	6.38±1.66	6.30±1.62	6.35±1.70	0.477
Neu%	60.35±9.77	60.79±8.95	58.60±8.56	59.21±9.84	0.421
Lym%	29.72±8.99	28.85±7.60	30.37±6.92	30.78±8.98	0.472

Group I: tiotropium 18ug once daily; Group II, budesonide/formoterol 160/4.5ug twice daily; Group III, budesonide/formoterol 320/9ug twice daily; Group IV, tiotropium 18ug once daily+ budesonide/formoterol 160/4.5ug twice daily.

BMI: body mass index; COPD: chronic obstructive pulmonary disease;

*: the number of exacerbations or hospitalizations in the preceding year;

FEV_1_: forced expiratory volume in one second; FVC: forced vital capacity; CVD: cardiovascular disease; DM: Diabetes mellitus; mMRC: modified British medical Research Council dyspnoea scale; CAT: COPD assessment test; CRP: C-reactive protein; IL-6: interleukin-6; IL-8: interleukin-8; SAA: serum amyloid; TNF-α: tumour necrosis factor-α; Fib: fibrinogen; WBC: white blood cell; Neu: neutrophil; Lym: lymphocyte.

Data are shown as mean ± SD, unless indicated otherwise.

^δ^: Nonormally distributed variables are shown as median (interquartile range).

### Systemic inflammation biomarkers

#### (1) CRP

Group I: the median baseline CRP level was 3.54 mg/L. The median CRP levels of subjects who completed the second, third and fourth visit were 3.87 mg/L, 4.46 mg/L and 4.05 mg/L, respectively, none of which with statistical difference compared with the baseline level (Table 2). Group II: the median baseline CRP level was 3.90 mg/L. The median CRP levels of subjects who completed the second, third and fourth visit were 3.79 mg/L, 3.32 mg/L (compared with baseline, *P*<0.05) and 3.41 mg/L (compared with baseline, *P*<0.05), respectively (Table 3). Group III: the median baseline CRP level was 3.69 mg/L. The median CRP levels of subjects who completed the second, third and fourth visit were 3.16 mg/L, 3.08 mg/L and 3.10 mg/L, respectively, all of which with statistical differences compared with the baseline level (Table 4). Group IV: the median baseline CRP level was 4.20 mg/L. The median CRP levels of subjects who completed the second, third and fourth visit were 3.48 mg/L, 3.68 mg/L and 3.27 mg/L, respectively, all of which with statistical differences compared with the baseline level (Table 5).

**Table 2:** Changes in circulating biomarkers, pulmonary function and CAT scores of subjects in group I during six months of therapy.

	Visit 1	Visit 2	Visit 3	Visit 4
n	83	75	68	65
CRP (mg/L) ^δ^	3.54(1.60, 5.32)	3.87(2.33, 6.33)	4.46(2.72, 7.46)	4.05(2.75, 5.90)
IL-6 (pg/mL) ^δ^	4.40(3.13, 7.29)	4.57(3.09, 7.87)	4.80(3.35, 8.12)	4.94(3.44, 8.32)
IL-8 (pg/mL)	11.54±6.82	10.15±4.78	11.09±6.23	10.37±4.83
SAA (mg/L) ^δ^	3.52(2.32, 10.00)	3.61(2.36, 7.96)	3.84(2.36, 7.93)	4.27(2.39, 10.99)
TNF-α (pg/mL)	10.39±3.19	10.71±3.02	10.77±3.13	10.70±2.86
Fib (g/L)	3.58±0.90	3.50±0.94	3.53±0.98	3.48±0.89
WBC (×10^9^/L)	6.67±1.66	6.18±1.72	6.28±1.75	6.06±1.77*
Neu (%)	60.35±9.77	61.77±9.20	61.75±9.00	62.10±8.50
Lym (%)	29.72±8.99	31.35±8.39	31.22±8.27	30.93±8.29
FEV_1_ (L)	0.88±0.34	ND	1.02±0.23*	1.00±0.22*
FEV_1_% predicted	37.73±9.97	ND	41.66±9.96*	41.28±10.25*
FVC (L)	2.21±0.72	ND	2.37±0.58*	2.33±0.54*
FEV_1_/FVC (%)	40.11±7.29	ND	45.12±12.15	45.02±12.32*
CAT score	16.29±5.14	11.29±2.14*	9.38±2.13*	8.65±1.64*

Group I: tiotropium 18ug once daily group; CAT: COPD assessment test; Visit 1: at the beginning of therapy; Visit 2: at the end of 1 month-therapy; Visit 3: at the end of 3 month-therapy; Visit 4: at the end of 6 month-therapy.

*: compared to baseline, *P* <0.05.

CRP: C-reactive protein; IL-6: interleukin-6; IL-8: interleukin-8; SAA: serum amyloid; TNF-α: tumour necrosis factor-α; Fib: fibrinogen; WBC: white blood cell; Neu: neutrophil; Lym: lymphocyte; FEV_1_: forced expiratory volume in one second; FVC: forced vital capacity; ND: not detected.

Data are shown as mean ± SD, unless indicated otherwise.

^δ^: Nonormally distributed variables are shown as median (interquartile range).

**Table 3:** Changes in circulating biomarkers, pulmonary function and CAT scores of subjects in group II during six months of therapy.

	Visit 1	Visit 2	Visit 3	Visit 4
n	79	75	70	67
CRP (mg/L) ^δ^	3.90 (2.97, 6.56)	3.79(1.77, 6.32)	3.32(1.44, 5.16)*	3.41(1.16, 4.69)*
IL-6 (pg/mL) ^δ^	4.50(3.09, 9.39)	4.20(2.87, 6.51)	3.99(2.43, 5.82)*	3.64(2.37, 6.20)*
IL-8 (pg/mL)	10.99±5.61	9.91±4.31	9.78±3.76	9.50±3.34
SAA (mg/L) ^δ^	4.06(2.32, 11.98)	3.52(2.18, 7.50)	3.86(2.57, 7.79)	3.89(2.70, 7.78)
TNF-α (pg/mL)	10.90±3.66	10.51±4.21	11.12±2.83	11.36±3.14
Fib (g/L)	3.49±0.76	3.40±0.80	3.20±0.67 *	3.29±0.71
WBC (×10^9^/L)	6.38±1.66	6.71±1.67	6.66±1.67	6.43±1.71
Neu (%)	60.79±8.95	60.34±9.30	60.83±8.89	62.35±8.56
Lym (%)	28.85±7.60	29.61±8.34	28.78±8.11	28.71±8.00
FEV_1_ (L)	0.82±0.27	ND	0.98±0.19*	0.98±0.18*
FEV_1_% predicted	39.08±10.78	ND	42.13±10.75*	41.99±10.99*
FVC (L)	1.89±0.65	ND	2.27±0.60	2.27±0.61
FEV_1_/FVC (%)	45.63±8.63	ND	45.92±13.59	46.32±14.10
CAT score	16.15±4.74	12.07±3.02*	10.01±2.76*	9.70±2.22*

Group II: budesonide/formoterol 160/4.5ug twice daily group; CAT: COPD assessment test; Visit 1: at the beginning of therapy; Visit 2: at the end of 1 month-therapy; Visit 3: at the end of 3 month-therapy; Visit 4: at the end of 6 month-therapy.

*: compared to baseline, P <0.05.

CRP: C-reactive protein; IL-6: interleukin-6; IL-8: interleukin-8; SAA: serum amyloid; TNF-α: tumour necrosis factor-α; Fib: fibrinogen; WBC: white blood cell; Neu: neutrophil; Lym: lymphocyte; FEV1: forced expiratory volume in one second; FVC: forced vital capacity; ND: not detected.

Data are shown as mean ± SD, unless indicated otherwise.

^δ^: Nonormally distributed variables are shown as median (interquartile range).

**Table 4:** Changes in circulating biomarkers, pulmonary function and CAT scores of subjects in group III during six months of therapy.

	Visit 1	Visit 2	Visit 3	Visit 4
n	80	77	66	64
CRP (mg/L) ^δ^	3.69(1.98, 5.21)	3.16(2.08, 4.10)*	3.08(1.83, 3.78)*	3.10(1.80, 3.86)*
IL-6 (pg/mL) ^δ^	4.55(3.11, 8.61)	3.72(2.48, 6.54)*	3.70(2.63, 4.98)	3.35(2.39, 5.43)*
IL-8 (pg/mL)	10.44±4.61	9.32±4.55	10.40±5.41	9.86±5.16
SAA (mg/L) ^δ^	4.12(2.66, 7.37)	3.84(2.42, 7.44)	4.55(2.78, 8.33)	4.21(2.47, 8.47)
TNF-α (pg/mL)	10.73±3.60	10.77±3.26	10.91±2.50	11.28±2.77
Fib (g/L)	3.50±0.84	3.37±0.83	3.23±0.61	3.22±0.78
WBC (×10^9^/L)	6.30±1.62	6.64±1.64	6.59±1.66	6.23±1.65
Neu (%)	58.60±8.56	60.04±9.72	60.32±9.72	59.75±10.32
Lym (%)	30.37±6.92	29.57±8.47	29.38±8.58	29.25±9.11
FEV_1_ (L)	0.93±0.27	ND	1.06±0.27*	1.08±0.26*
FEV_1_% predicted	42.41±12.43	ND	42.10±11.98*	42.67±12.02*
FVC (L)	2.21±0.65	ND	2.23±0.52	2.31±0.53*
FEV_1_/FVC (%)	43.59±8.32	ND	49.68±14.98*	50.68±15.09*
CAT score	15.40±4.19	9.95±2.05*	9.02±2.57*	8.72±2.14*

Group III, budesonide/formoterol 320/9ug twice daily group; CAT: COPD assessment test; Visit 1: at the beginning of therapy; Visit 2: at the end of 1 month-therapy; Visit 3: at the end of 3 month-therapy; Visit 4: at the end of 6 month-therapy.

*: compared to baseline, *P* <0.05.

CRP: C-reactive protein; IL-6: interleukin-6; IL-8: interleukin-8; SAA: serum amyloid; TNF-α: tumour necrosis factor-α; Fib: fibrinogen; WBC: white blood cell; Neu: neutrophil; Lym: lymphocyte; FEV_1_: forced expiratory volume in one second; FVC: forced vital capacity; ND: not detected.

Data are shown as mean ± SD, unless indicated otherwise.

^δ^: Nonormally distributed variables are shown as median (interquartile range).

**Table 5:** Changes in circulating biomarkers, pulmonary function and CAT scores of subjects in group IV during six months of therapy.

	Visit 1	Visit 2	Visit 3	Visit 4
n	82	74	64	60
CRP (mg/L) ^δ^	4.20(2.36, 6.40)	3.48(2.56, 5.48)*	3.68(2.68, 5.21)*	3.27(2.05, 4.53)*
IL-6 (pg/mL) ^δ^	5.34(3.06, 10.11)	4.47(3.11, 7.57)*	3.94(2.80, 7.27)*	4.00(2.60, 6.94)*
IL-8 (pg/mL)	11.41±5.84	9.87±4.29	9.94±3.57	10.46±3.17
SAA (mg/L) ^δ^	4.46(2.42, 12.22)	4.19(2.29, 7.86)	4.16(2.34, 7.80)	5.21(3.67, 8.93)
TNF-α (pg/mL)	11.37±4.19	10.96±2.99	11.87±3.19	12.10±3.70
Fib (g/L)	3.49±0.85	3.29±0.84	3.20±0.80	3.32±0.72
WBC (×10^9^/L)	6.35±1.70	6.51±1.63	6.39±1.68	6.11±1.68
Neu (%)	59.21±9.84	58.89±8.70	58.69±8.88	58.77±9.37
Lym (%)	30.78±8.98	30.91±7.83	31.20±8.19	30.90±8.75
FEV_1_ (L)	0.81±0.30	ND	1.07±0.20*	1.08±0.20*
FEV_1_% predicted	34.12±12.85	ND	44.36±12.22*	45.01±12.04*
FVC (L)	1.95±0.50	ND	2.41±0.65*	2.45±0.68*
FEV_1_/FVC (%)	41.51±11.15	ND	46.83±12.96*	46.85±12.72*
CAT score	15.77±4.91	9.32±2.47*	8.48±2.49*	7.93±1.65*

Group IV, tiotropium 18ug once daily+ budesonide/formoterol 160/4.5ug twice daily group; CAT: COPD assessment test; Visit 1: at the beginning of therapy; Visit 2: at the end of 1 month-therapy; Visit 3: at the end of 3 month-therapy; Visit 4: at the end of 6 month-therapy.

*: compared to baseline, *P* <0.05.

CRP: C-reactive protein; IL-6: interleukin-6; IL-8: interleukin-8; SAA: serum amyloid; TNF-α: tumour necrosis factor-α; Fib: fibrinogen; WBC: white blood cell; Neu: neutrophil; Lym: lymphocyte; FEV_1_: forced expiratory volume in one second; FVC: forced vital capacity; ND: not detected.

Data are shown as mean ± SD, unless indicated otherwise.

^δ^: Nonormally distributed variables are shown as median (interquartile range).

**Fig 2:**
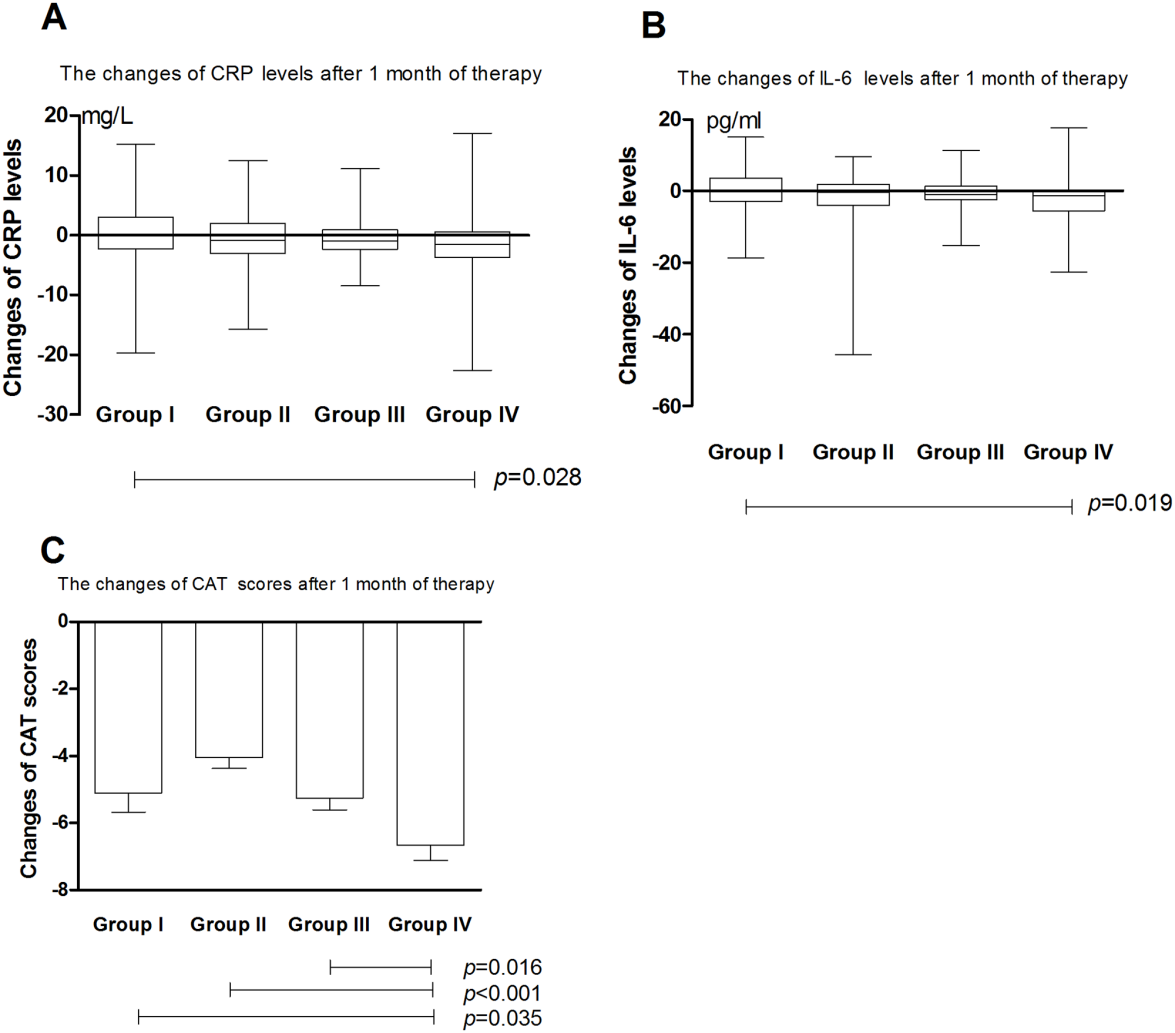
Treatment differences in terms of A) CRP, B) IL-6, and C) CAT Score after 1 month of therapy. Group I: tiotropium 18ug once daily; Group II, budesonide/formoterol 160/4.5ug twice daily; Group III, budesonide/formoterol 320/9ug twice daily; Group IV, tiotropium 18ug once daily+ budesonide/formoterol 160/4.5ug twice daily; CRP: C-reactive protein; IL-6: interleukin-6; CAT: COPD assessment test.

**Fig 3:**
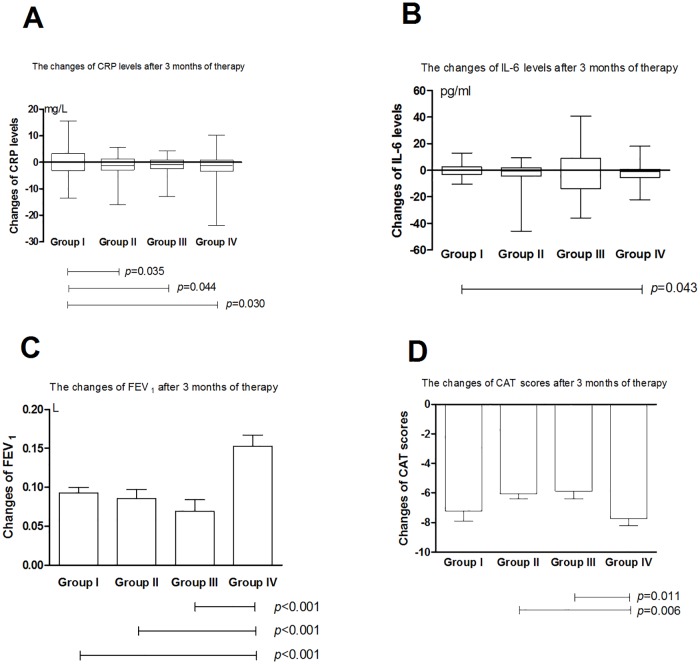
Treatment differences in terms of A) CRP, B) IL-6, C) FEV1 and D) CAT Score after 3 months of therapy. Group I: tiotropium 18ug once daily; Group II, budesonide/ formoterol 160/4.5ug twice daily; Group III, budesonide/formoterol 320/9ug twice daily; Group IV, tiotropium 18ug once daily+budesonide/formoterol 160/4.5ug twice daily; CRP: C-reactive protein; IL-6: interleukin-6; FEV1: forced expiratory volume in one second; CAT: COPD assessment test.

**Fig 4:**
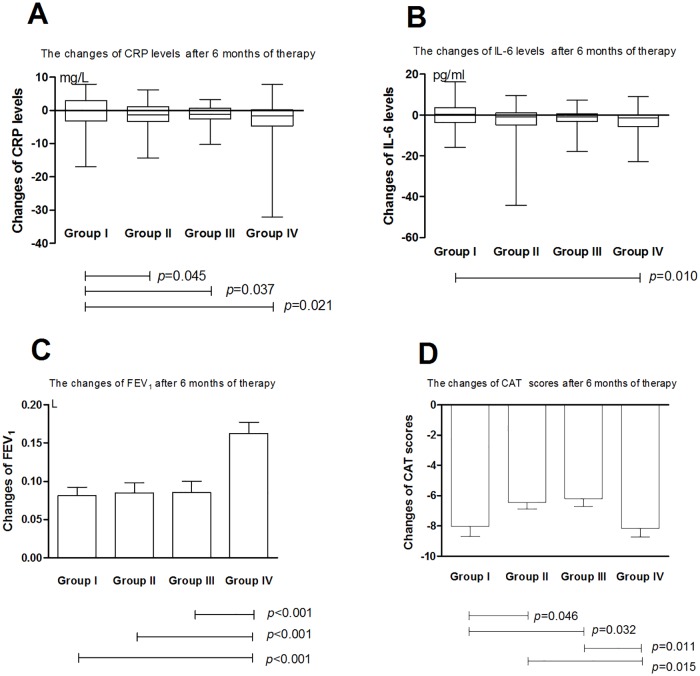
Treatment differences in terms of A) CRP, B) IL-6, C) FEV1 and D) CAT Score after 6 months of therapy. Group I: tiotropium 18ug once daily; Group II, budesonide/ formoterol 160/4.5ug twice daily; Group III, budesonide/formoterol 320/9ug twice daily; Group IV, tiotropium 18ug once daily+budesonide/formoterol 160/4.5ug twice daily; CRP: C-reactive protein; IL-6: interleukin-6;FEV1: forced expiratory volume in one second; CAT: COPD assessment test.

After 3-month and 6-month treatment, CRP level in Group II changed by a median (IQR) of -1.21 (-2.91, 1.19) mg/L and -1.25 (-3.29, 1.18) mg/L, respectively, both of which with statistical differences compared with group I. After 3-month and 6-month treatment, CRP level in Group III changed by a median (IQR) of -0.94 (-2.42, 0.82) mg/L and -1.13 (-2.55, 0.77) mg/L, respectively, both of which with statistical differences compared with group I. After 1-month, 3-month and 6-month treatment, CRP level in Group IV changed by a median (IQR) of -1.14 (-3.40, 0.96) mg/L, -1.30 (-3.39, 0.76) mg/L and -1.56 (-4.64, 0.22) mg/L, respectively, all of which with statistical differences compared with group I (Figs 2–4).

#### (2) IL-6

Group I: the median baseline IL-6 level was 4.40 pg/mL. The median IL-6 levels of subjects who completed the second, third and fourth visit were 4.57 pg/mL, 4.80 pg/mL and 4.94 pg/mL, respectively, none of which with statistical difference compared with the baseline level (Table 2). Group II: the median baseline IL-6 level was 4.50 pg/mL. The median IL-6 levels of subjects who completed the second, third and fourth visit were 4.20 pg/mL, 3.99 pg/mL (compared with baseline, *P*<0.05) and 3.64 pg/mL (compared with baseline, *P*<0.05), respectively (Table 3). Group III: the median baseline IL-6 level was 4.55 pg/mL. The median IL-6 levels of subjects who completed the second, third and fourth visit were 3.72 pg/mL(compared with baseline, *P*<0.05), 3.70 pg/mL and 3.35 pg/mL(compared with baseline, *P*<0.05), respectively (Table 4). Group IV: the median baseline IL-6 level was 5.34 pg/mL. The median IL-6 levels of subjects who completed the second, third and fourth visit were 4.47 pg/mL, 3.94 pg/mL and 4.00 pg/mL, respectively, all of which with statistical differences compared with the baseline level (Table 5).

After 1-month, 3-month and 6-month treatment, IL-6 level in Group IV changed by a median (IQR) of -0.93 (-5.13, 0.60) pg/mL, -1.14 (-5.37, 0.66) pg/mL and -1.50 (-5.56, 0.08) pg/mL, respectively, all of which with statistical differences compared with group I (Figs 2–4).

(3) The levels of IL-8, SAA, TNF-α, Fib and WBC of each group did not change significantly (shown in Tables 2–5).

### Pulmonary function

FEV_1_ of each group increased significantly after inhaled treatment (Tables 2–5). After 3-month and 6-month treatment, the mean increase of FEV_1_ in group IV was more than the other three groups (Figs 3 and 4).

### CAT score

CAT scores of each group decreased significantly after inhaled treatment (Tables 2–5). After 1-month treatment, the mean decrease of CAT scores in group IV was more prominent than the other three groups (Fig 2). After 3-month treatment, the mean decrease of CAT scores in group IV was more notably than group II and group III (Fig 3). After 6-month treatment, the mean decrease of CAT score in group I and IV was more distinct than group II and group III (Fig 4).

## Discussion

This is the first randomized (not double blinded) clinical trial to compare the effects of Tio and/or Bud/Form on systemic inflammation in stable COPD patients of group D. The study showed that a long-term treatment with Bud/Form alone or with Tio reduced circulating CRP levels in COPD patients of group D, while treatment with Tio alone did not. This result is similar to previous trials conducted by Sin et al [11]and Tang et al[14], which also showed that ICS and/or LABA reduced circulating level of CRP in COPD patients. These findings may explain the data of a cross-sectional study conducted by Pinto-Plata et al which showed that CRP levels were lower in users of ICS than in non-users in stable moderate-to-very severe COPD patients[12].

However, unlike our report, serum levels of CRP or IL-6 did not significantly change in inhaled fluticasone alone or in combination with salmeterol after 4 weeks of treatment[13]. Several important factors may facilitate to explain the discrepancies. First, the treatment duration was much longer (6 months vs. 4 weeks), which might lead to different outcomes. In our study, the changes of CRP levels were not significantly different at 1 month between Bud/Form group and Tio group. However, the levels of CRP were significantly reduced in Bud/Form alone or with Tio groups after 3-month and 6-month therapy. Therefore, the attenuation of underlying systemic inflammation by Bud/Form may be a progressive and lasting process. Second, different medications might also be an important factor accounting for this difference. In vitro experiments demonstrated that β 2-agonists varied in their suppressive effects on activated neutrophils[20]. Last, our participants did not take any corticosteroids prior to the study, which might eliminate the effects of corticosteroids on baseline systemic inflammation.

The origin of systemic inflammation in COPD is unclear. One potential explanation was that, somehow, pulmonary inflammation was “spilling over” into the systemic circulation[21]. Considering budesonide could attenuate the release and expression of IL-6 and IL-8 in bronchial epithelial cells[22] and block the release of cytokines and MMPs in COPD lung fibroblasts[23], it’s plausible that Bud/Form reduced systemic inflammation through attenuating airway and pulmonary inflammation in COPD. In addition, as IL-6 is a major signaling cytokine for CRP expression and other acute-phase proteins by hepatocytes[24], it’s possible that Bud/Form downregulated IL-6 production in the airways[22], which then reduced CRP expression by the liver. Moreover, Bud/Form could be absorbed systemically and then directly affected the hepatocytes. This might help to explain why the declines of CRP levels were accompanied with varying degrees of declines in IL-6 levels in our study.

Although the precise mechanism remained unknown, the finding of our study, which showed the intervention effect of Bud/Form on circulating CRP levels in COPD patients of group D, might have important clinical significance. COPD is a highly heterogeneous disease. There are significant differences in treatment options for patients with different clinical manifestations and severities. COPD patients of group D have many symptoms and a high risk of exacerbations. The first choice of therapy is ICS+LABA and/or LAMA[25]. But the guideline did not specify when an alternative monotherapy, and when the combination of medication[25]. The results of this study showed that, a long-term treatment with Bud/Form alone or with Tio can reduce circulating levels of CRP in COPD patients of group D compared with treatment with Tio alone. Since not all COPD patients have elevated systemic markers of inflammation[26], for those COPD patients with systemic inflammation in group D, Bud/Form might be a more appropriate choice.

Interestingly, all ICS-containing treatments reduced CRP levels 3 months after initiation of therapy, but only the combination of the Bud/Form with Tio significantly reduced CRP levels compared with Tio alone at 1 month, suggesting a modifying influence of the LAMA. These findings might explain the data of Cazzola M et al [27], which showed that adding Tio to ICS may provide benefits in symptomatic patients with severe-to-very severe stable COPD.

Reducing systemic inflammation in patients with COPD might have potential important clinical implications. It’s widely accepted that systemic inflammation is a key factor connecting COPD with its comorbidities[28–31]. And there is consistent evidence that these comorbiditie have a greater negative impact on the life qualities, exacerbation and mortality of COPD patients[32]. Therefore, reducing systemic inflammation might reduce the complications of COPD and then improve the prognosis of patients. Further follow-up cohort studies with larger samples would help to determine it.

Notably, although Bud/Form alone or with Tio reduced circulating CRP levels in COPD patients in our study, none of the four inhalation treatments showed the effect of intervening other systemic inflammation biomarkers including IL-8, SAA, TNF-α, Fib and WBC. Moreover, even after 6 months of treatment, the median values of CRP were all higher than 3mg/L in our study. And Previous population-based studies have reported the average CRP values were lower than 2.5 mg/L in adults who were 40 years of age [33, 34]. It meant that after 6 months of inhalation treatment, the CRP levels in COPD patients of group D might still be higher than non-COPD population. All of these findings suggested that Bud/Form could attenuate CRP levels in COPD patients of group D only to a limited extent and might be insufficient to restore it to the normal range. Therefore, further researches are needed to seek for the treatment of intervening systemic inflammation in patients with COPD.

Besides, the dosage of Bud/Form should also be highlighted for its value in clinical practice. An independent study showed that in Chinese patients with moderate to very severe COPD, fixed combination treatment with Bud/Form(160/4.5μg, two inhalations twice daily) resulted in clinically meaningful improvements in lung function, health-related quality-of-life, and COPD symptoms[35]. In our study, however, there was no statistical difference between the two treatments, Bud/Form (160/4.5μg, one inhalation twice daily) and Bud/Form (160/4.5μg two inhalations twice daily), in terms of the improvements in lung function, reducing symptoms and systemic inflammation. This result suggested that, for Chinese COPD patients of group D, increasing the dosage of Bud/Form (160/4.5μg, one inhalation twice daily) from one inhalation to two inhalations might not be helpful to increase the therapeutic effect. Considering the increase of the dosage adds the economic burden and increases the incidence of drug-related adverse events for patients[36, 37], the treatment of Bud/Form (160/4.5μg, one inhalation twice daily) could be considered for Chinese COPD patients of group D. More large-scale and long-term clinical trials should be conducted to confirm the result.

Although we see promising effect of Bud/Form attenuating circulating CRP levels in COPD patients of group D, there are some limitations in our present study. First, placebo treatment was not chosen for a control group. Since the subjects in our study were COPD patients of group D with obvious clinical symptoms and high risks, taking placebo alone was definitly improper for the ethical considerations. Second, interventions were open to participants, which was prone to introduce bias into the study. And the results of the CAT could have been biased by the non- blinded study design. However, the researchers who evaluated systemic inflammation biomarker levels and pulmonary functions were blind to the treatment. Besides, the data acquisition and data analysis were relatively independent. In this way, the generation of subjective bias was reduced. Third, the specificity of selected inflammation markers was not prominent enough, which were susceptible to be interfered by other diseases or factors. Therefore, we adopted strict exclusion criteria. Fourth, inflammatory biomarkers were not measured from lung samples due to ethical considerations and technical constraints, thus we could not further explore the possible origin of systemic inflammation and the mechanisms of drug intervention in COPD. In addition, there was lack of detailed screening for comorbidities. In our study, the establishment of comorbidities was based on patients’ history report, which might lead to missed diagnosis. For this reason, we did not further assess the relationship between systemic inflammation and comorbidities. Finally, the relationship between the treatment and acute exacerbation was not evaluated due to the relatively small number of cases and short follow-up period.

In summary, our clinical trial demonstrated that a long-term treatment with Bud/Form alone or with Tio can reduce circulating CRP levels in stable COPD patients of group D compared with treatment with Tio alone. The results highlighted the choice of individual therapy for COPD patients of group D. Longterm, large-scale, multicenter studies are needed in the future to determine whether Bud/Form can modify the progressive nature of chronic obstructive pulmonary disease.

## Supporting information

S1 File CONSORT checklist. (DOC)

S2 File Trial protocol in Chinese. (DOCX)

S3 File Trial protocol in English. (DOCX)

S4 File The data in the trial. (RAR)
