# Supplementary material for: Long-term treatment with budesonide/formoterol attenuates circulating CRP levels in chronic obstructive pulmonary disease patients of group D
Source: PLoS One. 2017 Aug 23;12(8):e0183300. doi: 10.1371/journal.pone.0183300 (PMC5568104; doi:10.1371/journal.pone.0183300)
Supplement: S2 File — (DOCX) [file pone.0183300.s002.docx]

课题研究方案

**研究题目：**噻托溴铵、布地奈德福莫特罗及其联合治疗对D组稳定期慢性阻塞性肺疾病患者系统性炎症影响

# 研究背景

慢性阻塞性肺疾病（chronic obstructive pulmonary disease，COPD）在全世界范围内是一种发病率和死亡率较高的重要疾病[[1](#_ENREF_1)]。预计至2020年COPD死亡率将升至第三位[[2](#_ENREF_2)]。

目前研究发现，COPD不仅是气道及肺的慢性炎症[[3](#_ENREF_3)],，并且是伴随肺外表现的低度、慢性的系统性炎症[[4](#_ENREF_4)]. 几种常见的已经被证实与COPD相关的系统性炎症指标包括：C反应蛋白（CRP）、白介素-6（IL-6）、白介素-8（IL-8）、血清淀粉样蛋白A（SAA）、肿瘤坏死因子-α (TNF-α)、纤维蛋白原(Fib)、以及白细胞（WBC）。很多观察性研究已经证实COPD系统性炎症与肺功能、动脉血氧分压、活动耐量、呼吸困难指数分级、临床转归、急性加重风险、死亡风险等密切相关[[5-7](#_ENREF_5)] 。COPD合并症（包括心血管疾病、肺癌、肺炎糖尿病、抑郁、恶液质、骨骼肌功能障碍及骨质疏松）[[8-10](#_ENREF_8)]。因此，干预系统性炎症有可能改善患者健康状况及疾病预后。

目前COPD全球倡议（GOLD）推荐长效抗胆碱药物（LAMA）、吸入性糖皮质激素（ICS）+长效β2受体激动剂（LABA）或其联合用药为D组稳定期COPD患者首选方案。这些药物除了扩张支气管外，也能减轻肺部炎症。但是这些药物对系统性炎症的干预作用还不明确。

因此，本研究拟采用前瞻性、随机、对照设计，探讨噻托溴铵、布地奈德/福莫特罗及联合方案对D组COPD稳定期患者的系统性炎症指标的影响及临床疗效，以期对COPD患者个体化治疗提供依据，并有助于我们进一步提高对该疾病的认识水平。

# 试验设计及流程

1.试验设计

本试验为随机、对照设计

2. 试验流程（见图1）

3. 样本量确定

本临床研究设立四组研究对象，按照1:1:1:1的比例分配。

一类错误α设为0.5，检验效能即1-β设为0.8，同时根据预实验及查阅文献得到受试者CRP的总体标准差σ为3，差值δ为1.5[[11](#_ENREF_11)]，用G*power3.1.7软件算得每组需要样本量为64例，考虑失访率不超过20%，最终确定每个试验组拟纳入样本量为80例，总共拟纳入样本量为320例。


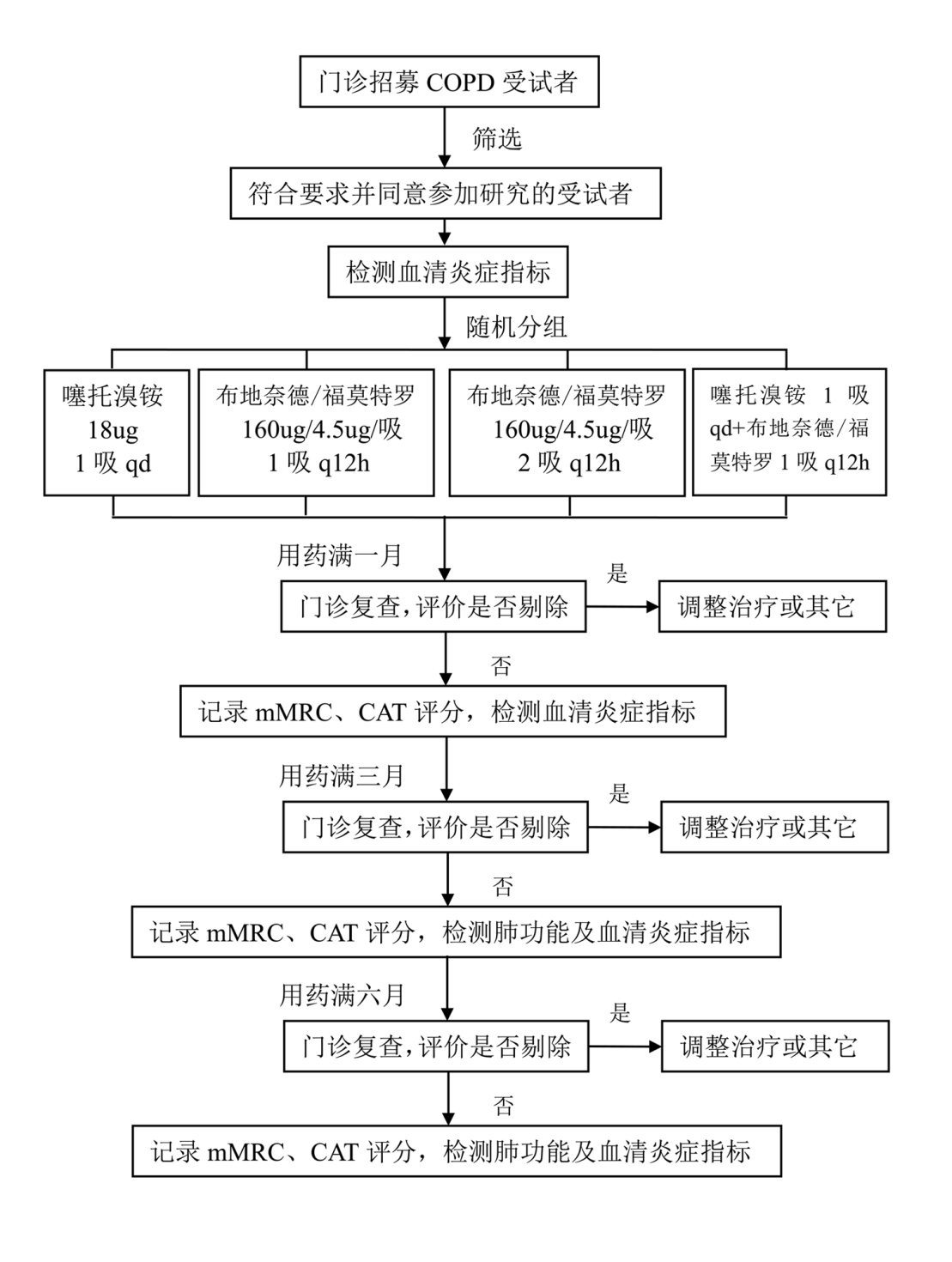


**图1.** 噻托溴铵、布地奈德/福莫特罗及其联合治疗对D组稳定期慢性阻塞性肺疾病患者系统性炎症指标影响的临床研究流程图

# 试验对象

# 1.试验对象

1.1 研究人群

于2015.1至2016.1在四川大学华西医院门诊招募符合要求的COPD患者。

1.1.1纳入标准

(1) 明确诊断为COPD稳定期且GOLD分组为D组[[12](#_ENREF_12)]；

(2) 年龄≥40岁；

(3) 就诊前1月内未出现急性加重。

1.1.2排除标准

(1) 就诊前1月内使用长效抗胆碱药物、长效β2受体激动剂、吸入性糖皮质激素及口服糖皮质激素、他汀类降脂药治疗；

(2) 就诊前1月内曾患有任何形式的感染性疾病者；

(3) 合并症状明显需要药物治疗的前列腺增生、膀胱颈狭窄者、窄角型青光眼者；

(4) 合并其它肺部疾病或系统性炎症升高的疾病（例如类风湿性关节炎，肝脏疾病，肾脏疾病，肿瘤或结核等）。

1.2 研究知情同意

对于根据上述标准纳入的患者，均告知研究内容并保证研究不会影响其诊疗过程，所采集的医学数据仅作科研用途，并保证患者隐私。签署知情同意后将患者列入研究对象。

试验干预措施及访视过程

1.干预措施

1.1试验用药

①噻托溴铵。商品名：思力华。生产企业：德国勃林格殷格翰公司。规格：18μg。进口药品注册标准JX20080017，进口药品注册证号H20050267，H20050268。

②布地奈德/福莫特罗粉吸入剂。商品名：信必可都保。生产企业：瑞典阿斯利康有限公司。规格：160ug/4.5ug/吸，60吸/支。进口药品注册标准：JX20040065，进口药品注册证号H20090773。

③沙丁胺醇吸入气雾剂。商品名：万托林。生产企业：西班牙葛兰素史克。规格：100ug×200揿。进口药品注册标准：JX20080307，进口药品注册证号：H200900514。

1.2随机分组给药

1.2.1试验分组

组I：噻托溴铵 18μg，1吸qd；

组II：布地奈德/福莫特罗 160ug/4.5ug/吸，1吸q12h.；

组III：布地奈德/福莫特罗 160ug/4.5ug/吸，2吸q12h；

组IV：噻托溴铵 1吸qd +布地奈德/福莫特罗 160ug/4.5ug/吸，1吸q12h。

四组受试者均给予沙丁胺醇吸入气雾剂，并告知其在紧急情况时可以用于缓解症状。

1.2.2随机化方法

采用完全随机设计。具体的随机化分配方法由研究人员依次编写好1～400的数字顺序表（此顺序即为以后进入临床试验的合格受试者的序号）。通过SPSS 19.0软件的Tansform、Compute variable及RV.UNIFORM函数编程产生随机数字，然后通过Tansform、Rank Cases函数编程产生随机数字的秩，最后通过Tansform、Recode into different variables函数产生试验编组，将受试者分配到组1至组4。以文件的形式一式两份保存随机分组表，并记录随机分组的产生方法、过程和分组结果。

1.3伴随用药

伴随用药是指受试者除了研究药物以外使用的其它药物（包括急救药物），分为允许用药和禁用药。

1.3.1允许使用的伴随用药

⑴ 急救使用的短效吸入型β2受体激动剂(SABA)沙丁胺醇，但需要详细记录给药剂量和时间；

⑵ 口服祛痰药如盐酸氨溴索片、桉柠蒎肠溶软胶、羧甲司坦、标准桃金娘油肠溶胶囊等，需要详细记录给药剂量和时间；

⑶ 如果有伴随疾病，任何不影响研究参数评估的必需治疗都是允许的。

1.3.2禁用的伴随用药

研究期间，禁止使用下列药物。一旦使用禁用药物，则受试者属于剔除病例。

⑴ 除噻托溴铵以外的其它抗胆碱能药，如异丙托溴铵；

⑵ 除福莫特罗以外的其它长效吸入型β2受体激动剂，如沙美特罗；

⑶ 口服β2受体激动剂如丙卡特罗、班布特罗等；

⑷ 除布地奈德以外的吸入或全身用糖皮质激素，如甲泼尼龙琥珀酸钠、泼尼松等；

⑸ 其它COPD治疗用药，如茶碱、氨茶碱、多索茶碱、罗氟司特等

⑹ 非甾体抗炎药，如双氯芬酸二乙胺、双氯芬酸钠等；

⑺ 他汀类药物，如阿托伐他汀、辛伐他汀等。

1.4其它伴随健康教育

所有患者在入组后均接受COPD常规健康教育，如避免吸烟、预防呼吸道感染及呼吸肌锻炼等。

2.试验访视过程（如表1）

2.1基线访视

⑴ 询问病史和人口学资料；

⑵ 常规体格检查；

⑶ 肺功能检查；

⑷ 症状评估，记录慢阻肺患者自我评估测试评分（CAT评分）[[13](#_ENREF_13)]；

⑸ 胸部CT检查；

⑹ 记录伴随疾病及其治疗；

⑺ 完成入选、排除标准的其它检查后签署知情同意书；

⑻ 慢性阻塞性肺疾病相关教育；

⑼ 系统性炎症标志物检测；

⑽ 按入组顺序编号，对照随机分组表，发放相应药物并交待药物使用方法和剂量。与患者随时保持联系，评价病情并判断是否剔除。

2.2访视2（用药足1月）

⑴ 询问症状，过去一月是否出现急性加重，是否出现其它症状或疾病；

⑵ 常规体检；

⑶ 记录用药情况；

⑷ 症状评估，记录CAT评分；

⑸ 记录不良事件；

⑹ 判断是否剔除；

⑺ 如不剔除，完成系统性炎症标志物检测并继续按试验方案给药。与患者随时保持联系，评价病情。

2.3访视3（用药足3月）

⑴ 询问症状，过去两月是否出现急性加重，是否出现其它症状或疾病；

⑵ 常规体检；

⑶ 记录用药情况；

⑷ 症状评估，记录CAT评分；

⑸ 记录不良事件；

⑹ 判断是否剔除；

⑺ 如不剔除，完成系统性炎症标志物检测及肺功能检测（用药前），并继续按试验方案给药。与患者随时保持联系，评价病情。

2.4访视4（用药足6月）

⑴ 询问症状，过去三月是否出现急性加重，是否出现其它症状或疾病；

⑵ 常规体检；

⑶ 记录用药情况；

⑷ 症状评估，记录CAT评分；

⑸ 记录不良事件；

⑹ 判断是否剔除；

⑺ 如不剔除，完成系统性炎症标志物检测及肺功能检测（用药前）。

表1. 患者资料收集流程表

| 时间  指标 | 基线访视  （0月） | 访视2  （1月） | 访视3  （3月） | 访视4  （6月） |
| --- | --- | --- | --- | --- |
| 是否合并AECOPD | √ | √ | √ | √ |
| CRP | √ | √ | √ | √ |
| IL-6 | √ | √ | √ | √ |
| IL-8 | √ | √ | √ | √ |
| Fib | √ | √ | √ | √ |
| TNF-α | √ | √ | √ | √ |
| SAA | √ | √ | √ | √ |
| WBC | √ | √ | √ | √ |
| FEV1 | √ |  | √ | √ |
| FVC | √ |  | √ | √ |
| FEV1/FVC | √ |  | √ | √ |
| CAT | √ | √ | √ | √ |

2.5病例剔除

进入药物干预和访视阶段的患者若出现以下情况则考虑剔除。

⑴ 未按试验要求应用研究药物（试验药物或安慰剂），依从性＜80%或者>120%者；

⑵ 违背试验给药方案；

⑶ COPD病情不能控制，出现COPD急性加重，不能继续原方案治疗者；

⑷ 受试者出现药物相关的严重不良反应，经评估不适合继续原方案治疗；

⑸ 随访期间受试者出现上述排除标准中的任何情况。

2.7病例脱离及处理

2.6落病例的定义

填写了知情同意书并筛选合格进入临床试验但没有完成临床试验方案所规定观察周期的受试者，称为脱落病例，包括以下几种情况：

① 受试者不愿意继续进行临床试验，向相关研究人员提出退出临床试验；

② 受试者失访。

2.7脱落病例的处理

对于脱落病例，研究者应采取电话、预约随访或者登门等方式，尽可能与受试者联系，询问理由，记录最后一次用药时间，并尽可能完成所能完成的评估项目。对于因过敏或其它不良反应、治疗无效而退出试验的病例，研究者根据受试者的实际情况，采取相应的治疗措施。同时，研究者应妥善保存脱落病例的有关试验资料，为进行全分析统计所需。

2.7中止及终止临床试验的标准

⑴ 试验过程中，出现与药物相关的严重不良反应；

⑵ 试验中发现临床试验方案有重大失误，难以评价药物效应；

⑶ 试验方案在实施中发生了重大偏差。

3.观察指标

3.1主要观察标准

主要观察指标包括治疗前和治疗1、3、6月后患者的系统性炎症指标，包括：

⑴ CRP；

⑵ IL-6；

⑶ IL-8；

⑷ Fib；

⑸ TNF-α；

⑹ SAA；

⑺ WBC。

血液标本送医院检验中心实验室，由统一标准方法进行测定。其标本处理、测定步骤和含量计算均分别按试剂盒说明书的操作规程进行。

3.2次要观察标准

次要观察指标包括：

⑴ 治疗前和治疗3、6月后患者的肺功能[[14](#_ENREF_14)] (包括FEV_1_、FEV_1_%预计值、FVC 、FVC占预计值%、FEV_1_/FVC)；

⑵ 治疗前和治疗1、3、6月后患者的症状评分（CAT评分）[[13](#_ENREF_13)]。

4.统计分析

4.1统计软件

随机数字的产生、统计描述和统计推断采用SPSS 19.0软件。

4.2统计分析内容

统计分析的内容主要包括：

⑴ 各组病例分布，总脱落率、剔除率比较；

⑵ 可比性分析：比较各治疗组人口学资料和其它基线值，以衡量各组的可比性；

⑶ 相关性分析： 依从性比较：比较各组患者是否按医嘱应用药物，是否应用方案中的禁用药物；

⑷ 有效性分析：比较不同访视阶段各组患者系统性炎症指标、症状评分及肺功能情况；

⑸ 影响疗效分析：如年龄、性别、吸烟等在用药前组间存在明显差异，或存在可能影响疗效的相关因素，疗效比较时这些因素应做协变量考虑，需做协方差分析；

4.3统计方法

主要包括：

⑴ 如有离群值，需进行专业和统计的分析，决定取舍；

⑵ 对未完成实验的病例分析：脱落病例和剔除病例应一一分析原因；

⑶ 所有计数指标采用百分比（%）表示。对所有计量资料进行正态性检验，其中符合正态分布的资料采用均值±标准差（mean±SD）表示，非正态分布的资料采用中位数（P_25_-P_75_）表示（P_25_、P_75_分别为第25个和第75个百分位数）。

⑷ 将研究对象随机分为4组，对研究对象的人口学资料、临床资料和结局指标进行组间比较，其中对服从正态分布的计量资料组间比较采用单因素ANOVA，并且以LSD法进行两两比较；非正态分布的计量资料组间比较采用Kruskal Wallis H法进行秩和检验，并且以Mann-Whitney U法进行两两比较；计数资料的组间比较采用χ^2^检验。p<0.05为差异有统计学意义。

⑸ 根据计量资料是否服从正态分布，分别采用Pearson法或Spearman法双变量相关分析系统性炎症指标与其他临床参数（如肺功能、症状评分、吸烟等）的关系。以系统性炎症指标为应变量，以相关的临床参数为自变量，采取向后筛选策略进行多元线性逐步回归分析，以剔除混淆因素。如果系统性炎症指标为非正态分布，多元线性逐步回归分析之前需作对数化处理。

⑹ 每一分组不同访视阶段治疗前后患者系统性炎症指标、症状评分及肺功能情况比较：资料如符合正态分布，采用配对样本*t*检验；如不符合正态分布，采用Wilcoxon法进行2个相关样本的秩和检验。

⑺不同访视阶段各个分组患者治疗前后系统性炎症指标、症状评分及肺功能变化情况比较：对服从正态分布的计量资料组间比较采用单因素ANOVA，并且以LSD法进行两两比较；非正态分布的计量资料组间比较采用Kruskal Wallis H法进行秩和检验，并且以Mann-Whitney U法进行两两比较。

参考文献

1. Kochanek K, Xu J, Murphy S. Mini no, AM and Kung, H.-C.(2011) Deaths: preliminary data for 2009. *National vital statistics reports: from the Centers for Disease Control and Prevention, National Center for Health Statistics National Vital Statistics System*; 54: 1-51.

2. Murray CJ, Lopez AD. Alternative projections of mortality and disability by cause 1990–2020: Global Burden of Disease Study. *The Lancet* 1997; 349: 1498-1504.

3. Hogg JC, Chu F, Utokaparch S, Woods R, Elliott WM, Buzatu L, Cherniack RM, Rogers RM, Sciurba FC, Coxson HO. The nature of small-airway obstruction in chronic obstructive pulmonary disease. *New England Journal of Medicine* 2004; 350: 2645-2653.

4. Gan W, Man S, Senthilselvan A, Sin D. Association between chronic obstructive pulmonary disease and systemic inflammation: a systematic review and a meta-analysis. *Thorax* 2004; 59: 574-580.

5. De Torres J, Cordoba-Lanus E, Lopez-Aguilar C, de Fuentes MM, de Garcini AM, Aguirre-Jaime A, Celli B, Casanova C. C-reactive protein levels and clinically important predictive outcomes in stable COPD patients. *European Respiratory Journal* 2006; 27: 902-907.

6. Garcia-Rio F, Miravitlles M, Soriano JB, Munoz L, Duran-Tauleria E, Sanchez G, Sobradillo V, Ancochea J. Systemic inflammation in chronic obstructive pulmonary disease: a population-based study. *Respir Res* 2010; 11: 63.

7. Dahl M, Vestbo J, Lange P, Bojesen SE, Tybjærg-Hansen A, Nordestgaard BG. C-reactive protein as a predictor of prognosis in chronic obstructive pulmonary disease. *American journal of respiratory and critical care medicine* 2007; 175: 250-255.

8. Thomsen M, Dahl M, Lange P, Vestbo J, Nordestgaard BG. Inflammatory biomarkers and comorbidities in chronic obstructive pulmonary disease. *American journal of respiratory and critical care medicine* 2012; 186: 982-988.

9. Reid MB, Li Y-P. Tumor necrosis factor-α and muscle wasting: a cellular perspective. *Respiratory research* 2001; 2: 269.

10. Liang B, Feng Y. The association of low bone mineral density with systemic inflammation in clinically stable COPD. *Endocrine* 2012; 42: 190-195.

11. Tang Y-j, Wang K, Yuan T, Qiu T, Xiao J, Yi Q, Feng Y-L. Salmeterol/fluticasone treatment reduces circulating C-reactive protein level in patients with stable chronic obstructive pulmonary disease. *Chinese Medical Journal (English Edition)* 2010; 123: 1652-1657.

12. GOLD Executive Committee. Global strategy for the diagnosis, management, and prevention of chronic obstructive pulmonary disease (Revised 2011). 2012. <http://www.goldcopd.com>.

13. Jones PW, Harding G, Berry P, Wiklund I, Chen WH, Kline Leidy N. Development and first validation of the COPD Assessment Test. *Eur Respir J* 2009; 34: 648-654.

14. Quanjer PH, Tammeling GJ, Cotes JE, Pedersen OF, Peslin R, Yernault JC. Lung volumes and forced ventilatory flows. Report Working Party Standardization of Lung Function Tests, European Community for Steel and Coal. Official Statement of the European Respiratory Society. *Eur Respir J Suppl* 1993; 16: 5-40.
