# Supplementary material for: Long-term treatment with budesonide/formoterol attenuates circulating CRP levels in chronic obstructive pulmonary disease patients of group D
Source: PLoS One. 2017 Aug 23;12(8):e0183300. doi: 10.1371/journal.pone.0183300 (PMC5568104; doi:10.1371/journal.pone.0183300)
Supplement: S3 File — (DOCX) [file pone.0183300.s003.docx]

**Study Protocol**

**Title：**The effects of tiotropium (Tio) and/or budesonide/formoterol (Bud/Form) on systemic inflammation biomarkers in stable COPD patients of group D

**1. Background**

Chronic obstructive pulmonary disease (COPD) is one of the leading causes of disability and death worldwide. It is the fourth most common cause of death in the world [[1](#_ENREF_1)] , and it is predicted to rise to the third position by the year 2020[[2](#_ENREF_2)]。

More and more studies have shown that COPD is not only a respiratory inflammatory condition[[3](#_ENREF_3)], but also one characterized by low-grade and chronic systemic inflammation with many extrapulmonary manifestations[[4](#_ENREF_4)]. Several biomarkers of systemic inflammation have been proved to be associated with COPD, including C-reactive protein (CRP), interleukin-6 (IL-6), interleukin-8 (IL-8), serum amyloid A (SAA), tumor necrosis factor-α (TNF-α), fibrinogen (Fib), and white blood cell (WBC). The systemic inflammation is associated with lung function, arterial oxygen tension, exercise capacity, degree of dyspnea, clinical outcome, risk of exacerbation, all-cause and COPD-related mortality[[5-7](#_ENREF_5)]. Moreover, the systemic inflammation is associated with increased risk of major comorbidities in COPD, including cardiovascular disease, lung cancer, pneumonia, diabetes mellitus, depression, cachexia, skeletal muscle dysfunction and osteoporosis[[8-10](#_ENREF_8)]. So attenuating systemic inflammation may have the potential to improve health status and prognosis of COPD patients.

The Global Initiative for Chronic Obstructive Lung Disease (GOLD) recommends that inhaled corticosteroid +long-acting β2-agonist and/or long-acting anticholinergic agents are first choices for COPD patients of group D. Few studies have been undertaken to evaluate whether those inhaled drugs have impact on the systemic inflammation in COPD.

Thus, a clinical trial will be conducted to investigate the effects of tiotropium (Tio) and/or budesonide/formoterol (Bud/Form) on systemic inflammation biomarkers in stable COPD patients of group D and improvements of symptoms and pulmonary function.

**2. Study design: A randomized controlled trial**

**2.1 Flow diagram of patients in the study (Fig 1)**


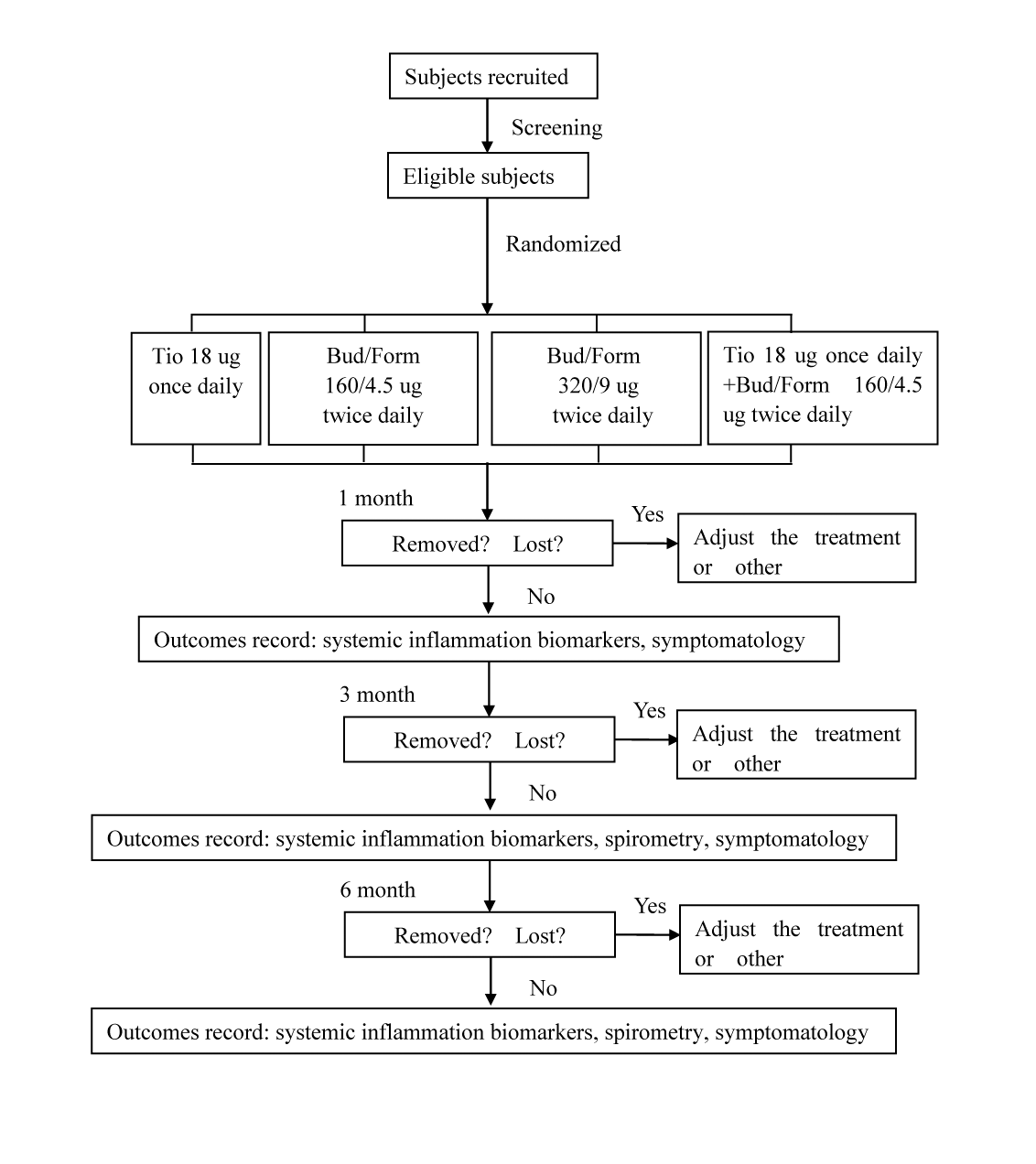
Fig 1 Flow diagram of patients in the study

**2.2 The estimation of the sample size**

CRP was selected as the reference for calculation of the estimated sample size. It was assumed that the mean difference in the change of CRP level was 1.5 mg/L between groups with an SD of 3 mg/L[[11](#_ENREF_11)], the level of significance was 0.05, the power of the test was 0.8, the lost follow-up rate was no more than 20%, and the calculated sample size was 80 per treatment group.

**3. Subjects**

Outpatients with a clinical diagnosis of stable COPD will be recruited from Jan 2015 to Jan 2016 in the West China Hospital of Sichuan University.

**3.1 Inclusion criteria**

(1) Confirmed COPD of group D by pulmonary physicians based on the 2011 GOLD guidelines[[12](#_ENREF_12)];

(2) Age ≥40 years;

(3) Absence of exacerbations[[12](#_ENREF_12)] for at least one month.

**3.2 Exclusion criteria**

(1) Patients used long-acting anticholinergic agents (LAMA), long-acting β2-agonist (LABA), oral or inhaled corticosteroid (ICS) in the previous one month;

(2) With any infection in the previous one month before study entry;

(3) With proved prostatic hyperplasia, bladder neck stenosis or narrow angle glaucoma;

(4) With other clinically significant lung diseases or complications which were associated with increasing systemic inflammation (e.g. rheumatoid arthritis, hepatic diseases, renal diseases, cancer or tuberculosis).

**4. Grouping and Intervention**

Eligible participants will be randomly assigned (1:1:1:1) using a computer-generated randomization list to one of four arms: group I, Tio 18ug once daily by Handihaler (Boehringer Ingelheim Pharma, Ingelheim, Germany); group II, Bud/Form 160/4.5ug/dose (Symbicort Turbuhaler; AstraZeneca, Sweden) one inhalation twice daily; group III, Bud/Form 160/4.5ug/dose two inhalations twice daily; group IV, Tio 18ug once daily plus Bud/Form 160/4.5ug/dose one inhalation twice daily.

All subjects will be allowed to take Terbutaline (Bricasol pressurized metered-dose inhale; AstraZeneca, Wuxi, China) as reliever during the study period. No other bronchodilator will be permitted to be used throughout the study.

**5. Follow-up**

The treatment duration is 6 months, with clinical visits at the end of the first month (visit 2), the third month (visit 3) and the sixth month (visit 4). Outcomes will be record during the follow-up (Table 1). During the treatment, subjects with any of the following conditions will be removed from the study: not taking medication as required; combined with acute exacerbations; occurring serious adverse events; combined with any one of the above exclusion criteria.

Table 1 Outcomes record during the follow-up.

| Time  Outcomes | Visit 1  （month 0） | Visit 2  （month 1） | Visit 3  （month 3） | Visit 4  （month 6） |
| --- | --- | --- | --- | --- |
| AECOPD? | √ | √ | √ | √ |
| CRP | √ | √ | √ | √ |
| IL-6 | √ | √ | √ | √ |
| IL-8 | √ | √ | √ | √ |
| Fib | √ | √ | √ | √ |
| TNF-α | √ | √ | √ | √ |
| SAA | √ | √ | √ | √ |
| WBC | √ | √ | √ | √ |
| FEV1 | √ |  | √ | √ |
| FVC | √ |  | √ | √ |
| FEV1/FVC | √ |  | √ | √ |
| CAT | √ | √ | √ | √ |

**6. Outcome measures**

6.1 Primary outcome measures: systemic inflammation biomarkers (including CRP, IL-6, IL-8, SAA, TNF-α, Fib, and WBC).

During each visit, fasting blood samples were collected in the morning. The samples were stored at -80°C until analyzed with standard hospital assays in the central laboratory.

6.2 Secondary outcome measures

(1) Spirometry [[13](#_ENREF_13)]: including forced expiratory volume in one second (FEV1), FEV1%pred, forced vital capacity (FVC), FVC%pred, FEV1/FVC.

(2) Symptomatology: COPD assessment test[[14](#_ENREF_14)].

**7. Statistical analysis**

All statistical analysis was performed using SPSS 19.0 (SPSS, Inc., Chicago, USA). Analyses will be conducted based on an intention-to-treat principle. Normally distributed data will be described as mean ± standard deviations (SD), whereas non-normally distributed data will be reported as medians (interquartile range) unless otherwise indicated.

For each visit of every group, changes from baseline in systemic inflammation biomarkers, lung function, and symptom scores will be analyzed using paired *t* tests or wilcoxon rank sum test, according to whether they meet the normal distribution.

For normally distributed variables, the differences of changes (post-treatment minus pre-treatment) between each groups will be analyzed using an analysis of variance (ANOVA) model. And the least significance difference test will be applied for pairwise comparison following ANOVA. For nonnormally distributed variables, the differences of changes between each group will be analyzed using Kruskal-Wallis test. And the Mann-Whitney U test applying for pairwise comparison will be followed.

**References**

1. Kochanek K, Xu J, Murphy S, AM M and Kung H. Deaths: preliminary data for 2009. *Natl Vital Stat Rep* 2011; 54: 1-51.

2. Murray CJ and Lopez AD. Alternative projections of mortality and disability by cause 1990–2020: Global Burden of Disease Study. *The Lancet* 1997; 349: 1498-1504.

3. Hogg JC, Chu F, Utokaparch S, Woods R, Elliott WM, Buzatu L, et al. The nature of small-airway obstruction in chronic obstructive pulmonary disease. *N Engl J Med* 2004; 350: 2645-2653.

4. Gan W, Man S, Senthilselvan A and Sin D. Association between chronic obstructive pulmonary disease and systemic inflammation: a systematic review and a meta-analysis. *Thorax* 2004; 59: 574-580.

5. De Torres J, Cordoba-Lanus E, Lopez-Aguilar C, de Fuentes MM, de Garcini AM, Aguirre-Jaime A, et al. C-reactive protein levels and clinically important predictive outcomes in stable COPD patients. *Eur Respir J* 2006; 27: 902-907.

6. Garcia-Rio F, Miravitlles M, Soriano JB, Munoz L, Duran-Tauleria E, Sanchez G, et al. Systemic inflammation in chronic obstructive pulmonary disease: a population-based study. *Respir Res* 2010; 11: 63.

7. Dahl M, Vestbo J, Lange P, Bojesen SE, Tybjærg-Hansen A and Nordestgaard BG. C-reactive protein as a predictor of prognosis in chronic obstructive pulmonary disease. *Am J Respir Crit Care Med* 2007; 175: 250-255.

8. Thomsen M, Dahl M, Lange P, Vestbo J and Nordestgaard BG. Inflammatory biomarkers and comorbidities in chronic obstructive pulmonary disease. *Am J Respir Crit Care Med* 2012; 186: 982-988.

9. Reid MB and Li Y-P. Tumor necrosis factor-α and muscle wasting: a cellular perspective. *Resp Res* 2001; 2: 269.

10. Liang B and Feng Y. The association of low bone mineral density with systemic inflammation in clinically stable COPD. *Endocrine* 2012; 42: 190-195.

11. Tang Y-j, Wang K, Yuan T, Qiu T, Xiao J, Yi Q, et al. Salmeterol/fluticasone treatment reduces circulating C-reactive protein level in patients with stable chronic obstructive pulmonary disease. *Chinese Med J (English Edition)* 2010; 123: 1652-1657.

12. GOLD Executive Committee. Global strategy for the diagnosis, management, and prevention of chronic obstructive pulmonary disease (Revised 2011). 2012. <http://www.goldcopd.com>.

13. Quanjer PH, Tammeling GJ, Cotes JE, Pedersen OF, Peslin R and Yernault JC. Lung volumes and forced ventilatory flows. Report Working Party Standardization of Lung Function Tests, European Community for Steel and Coal. Official Statement of the European Respiratory Society. *Eur Respir J Suppl* 1993; 16: 5-40.

14. Jones PW, Harding G, Berry P, Wiklund I, Chen WH and Kline Leidy N. Development and first validation of the COPD Assessment Test. *Eur Respir J* 2009; 34: 648-654.
